# Obstructive apneas induce early activation of mesenchymal stem cells and enhancement of endothelial wound healing

**DOI:** 10.1186/1465-9921-11-91

**Published:** 2010-07-06

**Authors:** Alba Carreras, Mauricio Rojas, Theodora Tsapikouni, Josep M Montserrat, Daniel Navajas, Ramon Farré

**Affiliations:** 1 Unitat de Biofísica i Bioenginyeria, Facultat de Medicina, Universitat de Barcelona -IDIBAPS, Barcelona, Spain; 2 CIBER Enfermedades Respiratorias, Bunyola, Spain; 3 Division of Pulmonary, Allergy and Critical Care Medicine, Department of Medicine, Emory University School of Medicine, Atlanta (GA), USA; 4 Servei Pneumologia, Hospital Clínic-IDIBAPS, Barcelona, Spain; 5 Institut de Bioenginyeria de Catalunya, Barcelona, Spain

## Abstract

**Background:**

The aim was to test the hypothesis that the blood serum of rats subjected to recurrent airway obstructions mimicking obstructive sleep apnea (OSA) induces early activation of bone marrow-derived mesenchymal stem cells (MSC) and enhancement of endothelial wound healing.

**Methods:**

We studied 30 control rats and 30 rats subjected to recurrent obstructive apneas (60 per hour, lasting 15 s each, for 5 h). The migration induced in MSC by apneic serum was measured by transwell assays. MSC-endothelial adhesion induced by apneic serum was assessed by incubating fluorescent-labelled MSC on monolayers of cultured endothelial cells from rat aorta. A wound healing assay was used to investigate the effect of apneic serum on endothelial repair.

**Results:**

Apneic serum showed significant increase in chemotaxis in MSC when compared with control serum: the normalized chemotaxis indices were 2.20 ± 0.58 (m ± SE) and 1.00 ± 0.26, respectively (p < 0.05). MSC adhesion to endothelial cells was greater (1.75 ± 0.14 -fold; p < 0.01) in apneic serum than in control serum. When compared with control serum, apneic serum significantly increased endothelial wound healing (2.01 ± 0.24 -fold; p < 0.05).

**Conclusions:**

The early increases induced by recurrent obstructive apneas in MSC migration, adhesion and endothelial repair suggest that these mechanisms play a role in the physiological response to the challenges associated to OSA.

## Background

Obstructive sleep apnea (OSA) is a prevalent disease affecting both children and adults. This sleep breathing disorder, caused by an abnormal increase in upper airway collapsibility, is characterized by recurrent events of airway obstruction, each finishing with the patient's unconscious arousal. These repetitive respiratory disturbances, which could appear more than once every minute in patients with severe OSA, induce increases in sympathetic activation, large negative intrathoracic pressure swings, hypoxia/reoxygenation events and disruption of sleep architecture. Extensive data in the literature prove that, in addition to immediate symptoms such as abnormal diurnal somnolence, OSA increases the mid- and long-term risk of metabolic dysfunctions and cardiovascular diseases [1,2]. Systemic inflammation and endothelial dysfunction triggered by recurrent hypoxia/reoxygenation have proved to be relevant processes as regards determining the consequences of OSA [3-5]. The susceptibility of distinct OSA patients to these consequences would, however, depend on the effectiveness of the individual homeostatic response to the challenges posed by the syndrome.

The main physiological response to the intermittent hypoxia and increased inspiratory efforts characteristic of OSA consists of the upregulation of well known signalling cascades that counteract oxidative stress and inflammation. Interestingly, data recently published on diseases distinct from OSA suggest that bone marrow-derived mesenchymal stem cells (MSC) circulating in peripheral blood could also contribute to the homeostatic response in OSA. Indeed, it has been shown that these stem cells play anti-inflammatory, anti-oxidative stress and endothelium-repairing roles via paracrine secretion of soluble factors [6,7]. The recent finding that the number of circulating MSC was acutely increased in a rat model of recurrent obstructive apneas [8] adds support to the hypothesis that MSC could be involved in the response to the injurious stimuli in OSA. In order to shed light on the potential role of MSC in this sleep breathing disorder, this study sought to investigate whether the blood serum of rats subjected to recurrent obstructive apneas mimicking OSA modulates basic mechanisms in the response to inflammation and endothelial damage: MSC migration and adhesion to endothelial cells and endothelial wound healing.

## Methods

### Application of recurrent obstructive apneas simulating OSA

**Figure 1:**
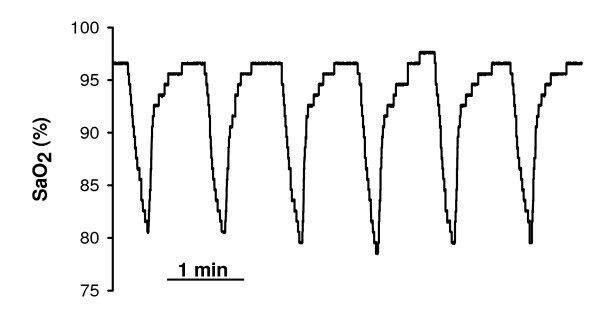
**Example of arterial blood oxygen saturation (SaO**_**2**_**) recorded in a rat during the application of recurrent airway obstructions**. The amplitude and time course of desaturations mimicked those typically observed in patients with obstructive sleep apnea.

This animal study was approved by the Ethical Committee for Animal Research of the University of Barcelona. Sixty Sprague-Dawley male rats (250-300 g) were intraperitoneally anaesthetized with urethane (1 mg/kg). Thirty rats were used as controls and 30 rats were subjected to recurrent airway obstructions at a rate of 60 apneas/hour for 5 hours, with each apnea lasting 15 seconds. The obstructive apneas were non-invasively applied by means of an electronically controlled nasal mask system recently described in detail by our group [8]. Arterial oxygen saturation was monitored by a pulse oxymeter (504; Critical Care Systems, Inc., Waukesha, WI) placed at the rat leg (Figure 1). After 5 h of recurrent obstructive apneas, 10-12 mL of blood from the carotid artery were collected in a serum separator gel tube and the rat was sacrificed by exsanguination. The serum was separated by centrifugation (1800 rpm, 20 min, room temperature) and frozen (-20°C) in aliquots for subsequent use. The sera of the 30 apneic rats and 30 control rats were randomly distributed for three different assays: migration, adhesion and wound healing assays using rat MSC and primary rat endothelial cells (10 apneic and 10 control rats for each assay).

### Mesenchymal stem cells

The study was performed on well-characterized Lewis rat marrow stromal cells kindly provided by Tulane Center for Gene Therapy [9]. Cells were cultured in MEM-alpha medium with L-glutamine and without ribonucleosides or deoxyribonucleosides (GIBCO, Gaithersburg, MD) supplemented with 20% fetal bovine serum (FBS; HyClone Cell Culture), 1% antibiotic-antimycotic (containing 10000 U/ml Penicillin G sodium, 10000 μg/ml Streptomycin sulfate, 25 μg/ml Amphotericin B as Fungizone in 0.85% saline (GIBCO, Gaithersburg, MD) and 2% L-glutamine (200 mM in 0.85% NaCl (GIBCO, Gaithersburg, MD). Cells were grown in an incubator (37°C, 5% CO_2_, 100% humidity). Subconfluent cells were dissociated with 0.25% trypsin and 1 mM Ethylene Diamine Tetraacetic Acid (EDTA) in Hanks' Balanced Salt Solution (GIBCO, Gaithersburg, MD) and subcultured at low density in new culture flasks. The differentiation potential of the MSC employed in this study was tested by culturing them in conventional adipogenic and osteogenic media for 21 days [8]. Positive differentiation into adipocytes and osteocytes was confirmed by staining the cells with Oil Red O and Alizarin Red S, respectively [8].

### Endothelial cell culture

Endothelial cell monolayers were obtained from anesthetized rats sacrificed by exsanguination through the carotid artery. A 2-cm long section of thoracic aorta was isolated and rinsed several times with Dulbecco's Phosphate Buffered Saline (DPBS) (Gibco™, Invitrogen, Carlsbad, CA, USA). The luminal artery surface was exposed to isolate the endothelial cells by incubation with collagenase II solution (1 mg/mL) (Gibco™, Invitrogen, Carlsbad, CA, USA) (37°C, 1 h) and centrifugation (1600 rpm, 10 min). After discarding the supernatant, cells were washed with DPBS and re-suspended in Dulbecco's modified Eagle's medium (DMEM) containing 1% (wt/vol) glucose (Gibco™, Invitrogen, Carlsbad, CA, USA), 10% inactivated fetal bovine serum (FBS) (Gibco™, Invitrogen, Carlsbad, CA, USA) and 0.5% antibiotics solution (streptomycin/penicillin solution 10,000U/ml) (Sigma Chemical Co., St. Louis, MO). Endothelial cells were cultured (37°C, 5% CO_2_, 100% humidity) by replacing the medium every 2-3 days until a cell monolayer was obtained (8-10 days).

### MSC migration assay

The chemotaxis and chemokinesis induced in MSC by the serum from control rats and from rats subjected to apneas (apneic serum) were assessed by inducing cell migration through the permeable membrane of transwells (6.5 membrane diameter, 8.0 μm pore filters; Corning Costar, Cambridge, MA). The upper side of the transwell membrane was coated with 0.1% (wt/vol) bovine gelatin (Sigma Chemical Co., St. Louis, MO) in DPBS for 1 h at 37°C. A suspension of 1.5 × 10^5 ^MSC in 190 μL of serum was placed in the upper compartment of the transwell and 1 ml of serum was placed in the lower compartment. The sera from 10 rats subjected to apneas and 10 control rats were used. Three transwell measurements were carried out for each pair of rat sera (apneic and control): 1) control serum in both the upper and lower compartment of the transwell (Control/Control), 2) apneic serum in both the upper and lower compartment of the membrane (Apnea/Apnea), and 3) apneic serum and control serum in the upper and lower transwell compartments, respectively (Apnea/Control). After 8 h of incubation of the transwell plates (5% CO_2 _at 37°C, 100% humidity), the upper side of the membrane was washed with cold DPBS. Using a cotton wool swab, the MSC remaining on the upper face of the membrane were removed and the cells on the lower side of the membrane were stained (May-Grünwald-Giemsa). The membrane was cut out with a scalpel, with the edges discarded, before being mounted on a micro slide glass, with the lower side on the top, and the image was digitized and stored (Eclipse TE2000-E, Nikkon; MetaMorph 7.6.1.0 software). The number of cells that migrated to the lower side of the membrane was counted by means of light microscopy, operated by an investigator who was blind to the types of sera present in each preparation. A normalized chemokinesis index was computed by dividing the number of cells counted in the Apnea/Apnea transwells by the number of cells counted in the Control/Control transwells. Similarly, a normalized chemotaxis index was computed by dividing the number of cells counted in the Apnea/Control transwells by the number of cells counted in the Control/Control transwells.

### MSC-endothelial adhesion assay

To assess the adhesion of MSC to endothelial cells when pretreated with control or apneic sera, they were first fluorescent-labelled with Vybrant CM-DiI (Gibco, Invitrogen, Carlsbad, CA, USA) at 6 μM for 20 min at 37°C and 15 min at 4°C (5% CO_2_, 100% humidity) and then washed with DPBS and re-suspended with complete medium. After labelling, MSC were pre-treated overnight with serum from 10 control rats or serum from 10 apneic rats and subsequently incubated on the endothelial cell monolayer for 6 h. The monolayer was then washed with medium and a fluorescent image was digitized and stored (Eclipse TE2000-E, Nikkon; MetaMorph 7.6.1.0 software). The MSC that remained adhered to the endothelial cells were counted by an investigator who was blind to the type of serum in each preparation. A normalized adhesion index was computed by dividing the number of cells counted by the mean value of counted cells in controls.

### Endothelial wound healing assay

A wound healing assay was used to investigate the effects of apneic serum on the repair of aortic endothelial cell monolayers. Briefly, 4 × 10^4 ^endothelial cells/well were seeded into 24-well plates and incubated (37°C, 5% CO_2_, 100% humidity) until they reached confluence. The endothelial cell monolayer of each well was scratch-wounded using a sterile 2-200 μL pipette tip (Eppendorf AG, Hamburg, Germany) and the debris was removed by washing with DPBS (Dulbecco's Phosphate Buffered Saline 1 × [-] CaCl_2_, [-] MgCl_2_; Gibco, Invitrogen, Carlsbad, CA, USA). The washing medium was subsequently removed and 300 μL/well of rat serum were used as the culture medium for the wounded endothelial monolayers. The well plate was then placed on the motorized stage of a microscope (Eclipse Ti, Nikon) equipped with a CCD camera (C9100, Hamamatsu) driven by MetaMorph 7.6.1.0 software. A microscope incubator (Life Imaging Services) maintained the whole system at 37°C, 5% CO_2 _and 100% humidity throughout the experiment. The endothelial wound healing process was assessed by automatically recording phase-contrast images of each well every 10 min from the beginning of the experiment up to 24 h of incubation. This wound healing assay was carried out using serum from 10 control rats and 10 apneic rats, in both cases with and without preconditioning the serum with MSC. Accordingly, a total of 40 wells were studied: non-conditioned serum and MSC-conditioned serum from each of the 10 apneic rats and 10 control rats. MSC-conditioned serum was obtained by culturing confluent MSC with rat serum for 48 h (24-well plate, 300 μL/well). At the end of the experiments, an investigator blind to the type of serum used in each well computed a wound closure index by comparing the initial and final (24 h) images of the endothelial wound. MetaMorph software was used to identify the initial and final limits of the wound. The closure index was computed as the increase in the wound's endothelial area, normalized to the mean increase in the case of the control serum.

### Statistical analysis

Data are presented as mean ± SEM. Comparisons between the different groups were carried out by means of the Student's t-test (when applicable) or the Mann-Whitney test. Statistical significance was established as p < 0.05.

## Results

**Figure 2:**
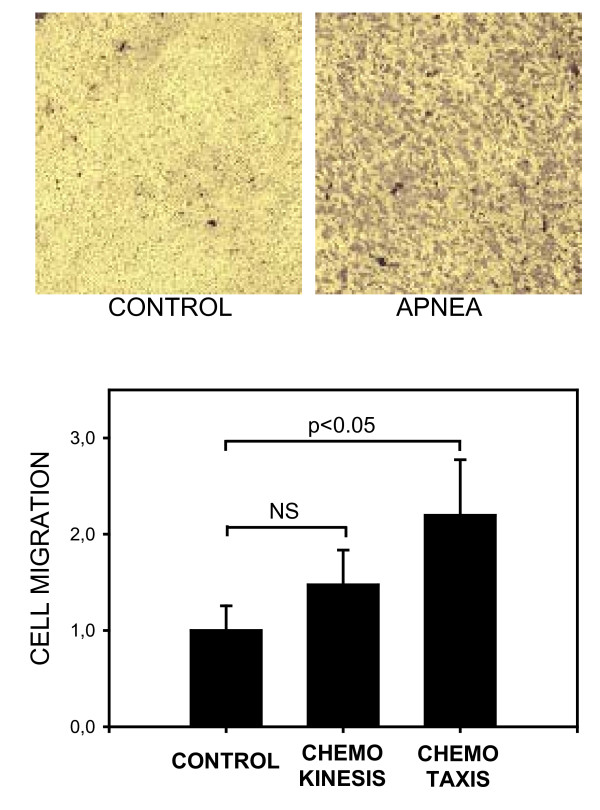
***Top*: Examples of the fields of view of transwell membranes, showing the stained MSC cells that migrated to the lower membrane side of transwells when the upper compartment contained control serum (left) and serum from rats subjected to recurrent obstructive apneas (right)**. Serum of control rats was placed in the lower transwell compartment in both cases. *Bottom*: Normalized indices for the migration of mesenchymal stem cells induced by the serum of control and apneic rats. Data are mean ± SEM. NS: non-significant (p > 0.05).

The serum of apneic rats increased the motility of MSC. As shown by the examples of transwell membrane images in Figure 2 (top), more cells migrated in the Apnea/Control transwell than in the Control/Control transwell. The bottom panel of this figure shows that the chemotaxis index for the serum of the apneic rats (2.20 ± 0.58) was significantly higher than that for the control serum (1.00 ± 0.26; p < 0.05). In contrast, the increase observed in the chemokinesis index when comparing the serum from apneic rats and the control serum was not statistically significant.

**Figure 3:**
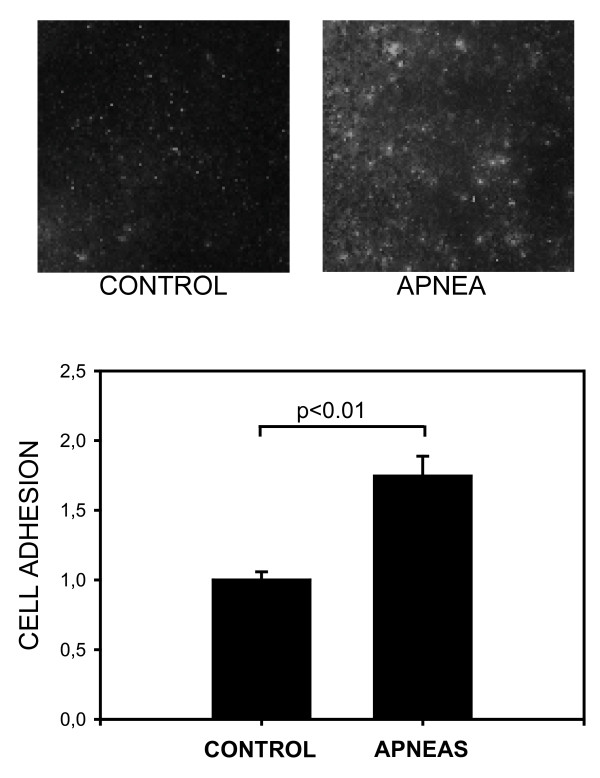
***Top*: Examples of fields of view of the fluorescence-labelled MSC adhered to cultured endothelial cells when incubated in control serum (left) and in serum from rats subjected to recurrent obstructive apneas (right)**. *Bottom*: Normalized index for the adhesion of MSC to endothelial cells in control rat serum and in serum from rats subjected to recurrent obstructive apneas. Data are mean ± SEM.

MSC exhibited significantly more adhesion to the monolayer of cultured endothelial cells when incubated in apneic rat serum as compared to control rat serum. Figure 3 (top) illustrates that more labelled-MSC adhered to the endothelial monolayer in the case of the apneic serum. This figure's bottom panel shows that the MSC-endothelial adhesion index was significantly higher in apneic serum than in control serum: 1.75 ± 0.14 and 1.00 ± 0.06, respectively (p < 0.01).

**Figure 4:**
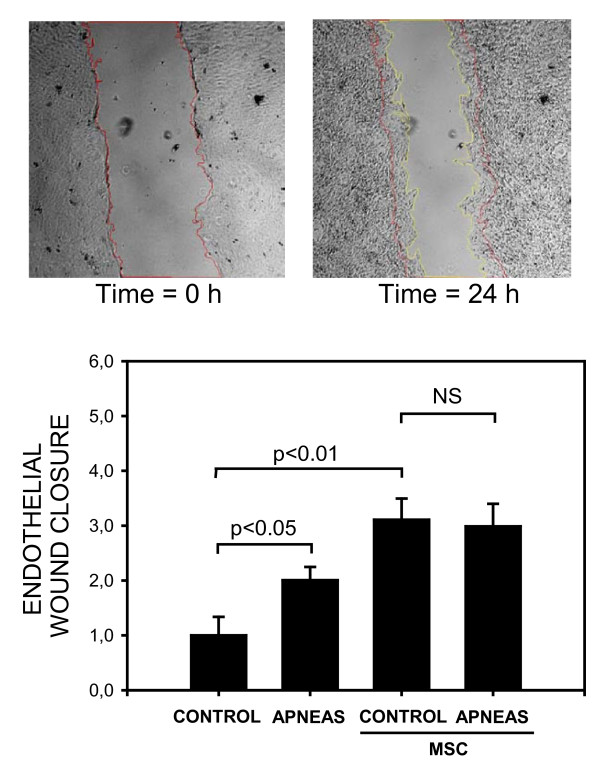
***Top*: Images of a representative example of an endothelial wound healing test showing the wound at the beginning of the experiment (left) and 24 h after the culture medium was replaced by apneic rat serum (right)**. Red and yellow lines indicate the initial and final wound borders. *Bottom*: Normalized index for wound closure when the endothelial cells were cultured in medium consisting of serum of control rats and of rats subjected to recurrent obstructive apneas, with or without preconditioning with MSC. Data are mean ± SEM.

As shown in Figure 4, endothelial wound healing was significantly increased when the injured monolayer was cultured with serum from rats subjected to recurrent apneas as compared with culture in control serum (2.01 ± 0.24 *vs *1.00 ± 0.34; p < 0.05). Fig. 4 also shows that preconditioning the control and apneic rat sera with MSC resulted in an increase in the endothelial wound closure index (3.11 ± 0.39 and 2.99 ± 0.41, respectively; p < 0.01 in both cases).

## Discussion

In this work we have assessed whether MSC could play a role in the physiological response to the injurious stimuli that characterize OSA and cause the cardiovascular consequences of this sleep breathing disorder. We focused our attention on basic mechanisms that potentially contribute to the repair of endothelial damage. The results obtained in this acute animal model study show that short-term recurrent obstructive apneas mimicking OSA triggered an early activation of MSC: specifically, an increase in the mobility of these stem cells and in their adhesion to endothelial cells. Moreover, it was also found that the serum of apneic rats improved endothelial wound healing and that MSC could contribute to this enhanced repair.

The potential protective or therapeutic role of MSC in various human diseases [10] and animal models has been extensively investigated for non-respiratory diseases [11-13], as well as for several respiratory pathologies [14-17]. It has been proven that, in addition to their capacity for tissue regeneration by homing at the injured tissue and differentiating onto damaged cell phenotypes, MSC secrete soluble mediators which can immuno-modulate inflammation and anti-oxidative cascades improving repair of vascular endothelium [6,7]. Only a very few studies, however, have focused on the potential role of stem cells in OSA, although there have been recent reports of the detection of circulating endothelial progenitor cells in patients [18-20] and circulating MSC in a rat model [8]. Accordingly, to our knowledge this is the first work studying how the stimuli characterizing OSA activate MSC responses and thus potentially contribute to the response to inflammation and the enhancement of vascular repair.

The methods used in the present study consisted of an *in vivo *rat model to obtain serum from control rats and from rats subjected to apneas and *in vitro *techniques to compare the effects of these sera on MSC and endothelial cell properties. One advantage of the animal model used in this study is that by non-invasively applying recurrent obstructive apneas in healthy animals the rats were subjected to periodic stimuli very similar (in magnitude, duration and frequency) to the ones experienced by patients with OSA: strenuous breathing efforts against a closed airway and hypoxemic events [21,22]. This acute model allowed us to study the early effects of these injurious stimuli while avoiding other confounding factors found in OSA patients that also cause inflammation and endothelial dysfunction (metabolic syndrome, obesity, hypertension, etc) [1-5]. The methodology employed in this short-term study may be also useful to investigate the chronic effects of long-term recurrent obstructive apneas in a chronic animal model [21]. Specifically, to investigate potential adaptation mechanisms in response to a long-term challenge as in OSA patients and to study the role of endothelial progenitor cells in vascular repair. The *in vitro *methodology used to investigate the effects of control and apneic rat sera on MSC and endothelial cells is widely reported in the literature. Indeed, the transwell setting for assessing chemotaxis and chemokinesis has previously been used to study MSC migration in response to different biochemical stimuli [23] and the fluorescent-staining method employed for assessing the adhesion of MSC to endothelial cell monolayers has been used in both in vivo and in vitro studies [24]. Moreover, the endothelial wound healing assay used in the present study is frequently encountered in research on endothelial repair mechanisms in vitro [25,26]. Interestingly, these *in vitro *methods are readily applicable in future studies to investigate whether the serum of OSA patients, before and after CPAP therapy, activates MSC and enhances endothelial repair when compared with serum from healthy controls.

Our experimental setting was designed to assess the early effects on MSC and endothelial cells induced by the serum of rats subjected to recurrent airway obstructions. Accordingly, the changes observed in migration, adhesion and wound healing were exclusively caused by the soluble factors released into the animals' serum as a result of subjecting them to the breathing stimulus mimicking OSA. This approach allowed us to identify the effects of soluble factors in blood from the effects of the other potentially important stimuli also experienced by these cells in OSA patients, such as intermittent hypoxia due to the recurrent changes in arterial oxygen saturation. The specific effects of intermittent hypoxia on cultured MSC and endothelial cells in OSA remain mostly unknown, given the technical difficulty of adequately applying, at the cell level, the high-rate of oxygen pressure changes mimicking OSA [27]. As regards the design of this study, it should also be pointed out that the experiments to assess the effects of rat serum on MSC-endothelial adhesion and on endothelial wound healing did not allow us to differentiate between the specific serum effects on each type of cell separately. This limitation does not, however, affect the aim of this study as these two types of cells are simultaneously exposed to the same blood serum in patients.

The blood serum of rats acutely subjected to recurrent obstructive apneas was chemoattractant for MSC (Figure 2). This could explain the finding in a previous study on a similar rat model that the number of MSC circulating in peripheral blood increased in apneic rats when compared with controls [8]. Accordingly, the soluble factors released into the serum as a rat's physiological response to the obstructive stimulus would be sensed by the MSC in the bone marrow (and in other tissues which are also reservoirs of these cells) and would induce their mobilization from their original niche to the bloodstream. In fact, this interpretation is consistent with the generally accepted hypothesis that a gradient of soluble factors secreted into the bloodstream is one of the mechanisms by which injured tissue induces the mobilization and recruitment of MSC. Although the exact mechanism triggering MSC mobilization is not known in detail, there is evidence to suggest that various growth factors and inflammatory cytokines characterizing inflammation in OSA (e.g. IL1-β and TNF-α) contribute to MSC migration [23].

Once released into the blood, circulating MSC migrate and home at the injured organ via a dynamic process similar to that of neutrophils in response to inflammation [28]: transient adhesion to endothelial cells, rolling, firm adhesion to the endothelium and transmigration into the tissues where MSC participate in the repair of the damaged tissue [29]. The enhancement observed in the adhesion of MSC on to endothelial cells (Figure 3) when cultured with apneic serum could be caused by the higher levels of pro-inflammatory cytokines in apneic serum. Effectively, IL1-β and TNF-α, which increase in the serum of animals in OSA models and in that of patients with this sleep disorder, have proved to increase the adhesion of MSC to cardiac endothelial cells both *in vivo *and *in vitro *[24]. Increased MSC-endothelial adhesion, together with the increase in MSC migration capacity (Figure 2) observed when mesenchymal stem cells were acutely exposed to the serum of rats subjected to recurrent apneas, suggests that OSA could facilitate a crucial step in tissue repair: the homing of MSC on the endothelium and injured sub-endothelial tissues.

A remarkable finding in this study was that, when compared with controls, the serum of rats subjected to recurrent obstructive apneas enhanced the wound closure of endothelial cell monolayers (Figure 4). Accordingly, the injurious stimuli in OSA (namely, intermittent hypoxia and strenuous breathing) would also trigger a response to enhance the repair of the injured endothelium. Whereas most of the published literature on vascular dysfunction in OSA is focused on the deleterious effects caused by blood soluble factors on the endothelium, little is known about the potential repair mechanisms triggered by injurious OSA stimuli. As endothelial wound healing is strongly enhanced by vascular endothelial growth factor (VEGF) [26] and the stimulus of OSA promotes an increase in blood VEGF [18], the observed increase in wound closure when culturing endothelial cells with apneic serum could be attributed to VEGF. In this respect, it is interesting to note that one potential source of the VEGF in the blood of apneic rats could be the circulating MSC induced by recurrent obstructive apneas [8]. Indeed, it has been shown that MSC express VEGF in response to a hypoxic stimulus [25]. It should be noted, however, that, regardless of the specific potential role of VEGF, MSC *per se *secrete factors that promote endothelial repair [30]. In line with this interpretation, we found that acutely preconditioning rat serum with MSC was sufficient to increase wound closure in both apneic and control sera (Figure 4). The fact that this increase was higher when the sera were preconditioned with MSC than when using apneic serum with no MSC preconditioning could be explained by the fact that the concentration of MSC in MSC-preconditioned sera was lower than in the circulating blood of apneic rats [8].

## Conclusions

This animal model study suggests that bone marrow-derived MSC could play a role in the physiological response to counterbalance the pro-inflammatory, oxidative stress and endothelial dysfunction mechanisms that lead to the middle- and long-term consequences of OSA.

## Competing interests

The authors declare that they have no competing interests.

## Authors' contributions

The conception and scientific direction of this work was undertaken by RF. Animal experimentation was carried out by AC and TS. Data processing and statistical analysis was undertaken by AC, TS, and JMM. AC, MR, TS, JMM, and DN participated in the discussion of the results and contributed to the manuscript draft. All authors read and gave critical input to this manuscript.
